# How does the knowledge environment shape procurement practices for orthopaedic medical devices in Mexico?

**DOI:** 10.1186/s12911-016-0324-1

**Published:** 2016-07-08

**Authors:** Myriam Lingg, Kaspar Wyss, Luis Durán-Arenas

**Affiliations:** University of Basel, Petersplatz 1, 4003 Basel, Switzerland; Swiss Tropical and Public Health Institute, Swiss Centre for International Health, Socinstrasse 57, 4051 Basel, Switzerland; National Autonomous University of Mexico, Medical faculty, Circuito Interior, Ciudad Universitaria, Av. Universidad 3000, CP 04510 Mexico, Mexico; Centre for Mexican Studies in the United Kingdom, London, WC2R 2LS UK

**Keywords:** Medical devices, Procurement, Healthcare systems, Orthopaedic, Knowledge management

## Abstract

**Background:**

In organisational theory there is an assumption that knowledge is used effectively in healthcare systems that perform well. Actors in healthcare systems focus on managing knowledge of clinical processes like, for example, clinical decision-making to improve patient care. We know little about connecting that knowledge to administrative processes like high-risk medical device procurement. We analysed knowledge-related factors that influence procurement and clinical procedures for orthopaedic medical devices in Mexico.

**Methods:**

We based our qualitative study on 48 semi-structured interviews with various stakeholders in Mexico: orthopaedic specialists, government officials, and social security system managers or administrators. We took a knowledge-management related perspective (i) to analyse factors of managing knowledge of clinical procedures, (ii) to assess the role of this knowledge and in relation to procurement of orthopaedic medical devices, and (iii) to determine how to improve the situation.

**Results:**

The results of this study are primarily relevant for Mexico but may also give impulsion to other health systems with highly standardized procurement practices. We found that knowledge of clinical procedures in orthopaedics is generated inconsistently and not always efficiently managed. Its support for procuring orthopaedic medical devices is insufficient. Identified deficiencies: leaders who lack guidance and direction and thus use knowledge poorly; failure to share knowledge; insufficiently defined formal structures and processes for collecting information and making it available to actors of health system; lack of strategies to benefit from synergies created by information and knowledge exchange. Many factors are related directly or indirectly to technological aspects, which are insufficiently developed.

**Conclusions:**

The content of this manuscript is novel as it analyses knowledge-related factors that influence procurement of orthopaedic medical devices in Mexico. Based on our results we recommend that the procurement mechanism should integrate knowledge from clinical procedures adequately in their decision-making. Without strong guidance, organisational changes, and support by technological solutions to improve the generation and management of knowledge, procurement processes for orthopaedic high-risk medical devices will remain sub-optimal.

## Background

Healthcare systems are knowledge intensive environments [1], where knowledge is a resource that must be efficiently managed [2]. “Knowledge management” and the “system thinking approach for systems’ knowledge” are systematic approaches to identifying, capturing, developing, sharing, and efficiently using knowledge [3, 4]. When healthcare systems take these approaches, resources like knowledge are used more efficiently [5–9]. Many knowledge frameworks exist and they encompass different strategies [10] to improve the systematic handling of knowledge and potential knowledge within systems [11]. In healthcare systems, stakeholders are concerned, for example, with knowledge from clinical procedures. This knowledge is created by processing different types of information, and is derived from health data, as well as clinical data, which includes (i) patient-related information, and (ii) management information bearing on processes and outcomes, such as the health status of a population [12].

In healthcare systems actors focus on managing knowledge of clinical procedures like clinical decision-making to improve patient care [13–16] and activities to assure healthcare worker and patient safety [17] (e.g. healthcare working conditions that influence patient outcomes). Using this knowledge effectively and efficiently requires a substantial understanding of factors determining its management. In the general theory of knowledge management the understanding of these factors (success or context factors) varies [10] but can be grouped along four dimensions. These dimensions originated from a study comparing 160 knowledge management frameworks and describing these dimensions as [11]: people (culture, people skills, and leadership); organisation (processes and structures); management (strategy, goals, and measurement); and, information technology (infrastructure and applications).

In healthcare systems, knowledge of clinical processes is an important resource across all stages of healthcare delivery (clinician, care provider facility, social security system, regulation, etc.) [18]. Understanding the management of knowledge in the context of these dimensions and across different stakeholders involved is necessary to solve or prevent problems related to knowledge. For instance, when organisations have to manage complaints and adverse events of medical devices, they must consider more than just organisational factors (processes, structures, etc.); they also need to engage relevant stakeholders working out strategies to prevent problems influencing clinical procedures and affecting healthcare worker and patient safety [17]. A complaint is a complication occurring in the course of pre- or intra-operative procedures like, for example, the positioning of a cap liner into the cap due to surgical technique, accompanying instruments or not visible damages to the liner. An adverse event is an undesirable occurrence for a patient and associated with the use of a medical device that requires extra treatment or the removal of an implanted medical device. For instance, a few years ago several removals of specific breast implants (quality of used material) and hip resurfacing implants (metal debris damaging bone) were necessary [19, 20].

These products are high-risk medical devices (HRMD) as they are highly regulated because they remain in the patients’ body [21]. Examples for HRMDs are those used in reconstructive surgery (breast implants, hip or knee implants) or in the treatment of diseases (coronary stents). Post-market surveillance plays an important role and encompasses the monitoring of the safety and effectiveness of medical devices once they are on the market and used in clinical settings [22]. This is an important function within a healthcare system because HRMDs often remain in the patient body. Healthcare systems and healthcare providers must integrate all four dimensions into their processes for capturing, developing, sharing, and effectively using knowledge and for building administrative frameworks [23, 24]. The contribution of information technology in order to manage big data across different levels of an organisation and healthcare system is significant.

Adequately managed knowledge can support administrative processes, such as procurement [23]. Procurement decision-making determines the devices and accompanying services used for the treatment of patients, and is knowledge-intensive [25]. Procuring HRMDs is a process in which administrations or procurement agents use certain information from various parties to inform purchasing decisions. Based on information it is the goal of procurement to purchase goods that have an optimal combination of high quality and low price [26]. The ways the health system or social security system manages knowledge will shape the way knowledge is used by procurement. The administrator or agent may only use rigid information about acquisition price and product specifications. Little is published about how knowledge of clinical procedures or information related is used in relation to administrative processes like procurement [26, 27].

### Purpose

This research is part of a larger study to improve the understanding of the connection between procurement processes for orthopaedic HRMDs in Mexico and clinical procedures. In our previous study we observed that in Mexico, mutual knowledge support (e.g. use of knowledge from arthroplasty registries) does not adequately benefit procurement and clinical procedures of orthopaedic HRMDs. In Mexico, orthopaedic speciality belongs to a concept of high level care attention and studies reported that high level care attention is still in need of being strengthened [28]. The role played by procurement is important because it provides clinicians with products and services. Previous research about public procurement in Mexico focused on one of the social security institutes providing an action plan for procurement officers, information systems and supplier performance [29]. The aim of our study is to analyse knowledge-related factors that influence procurement of orthopaedic HRMDs in Mexico and is governed by three objectives:

- Analyse factors of managing knowledge of clinical procedures.
- Assess the role of this knowledge and in relation to procurement of orthopaedic medical devices.
- Determine opportunities to improve the situation.

## Methods

### Study framework

**Fig. 1:**
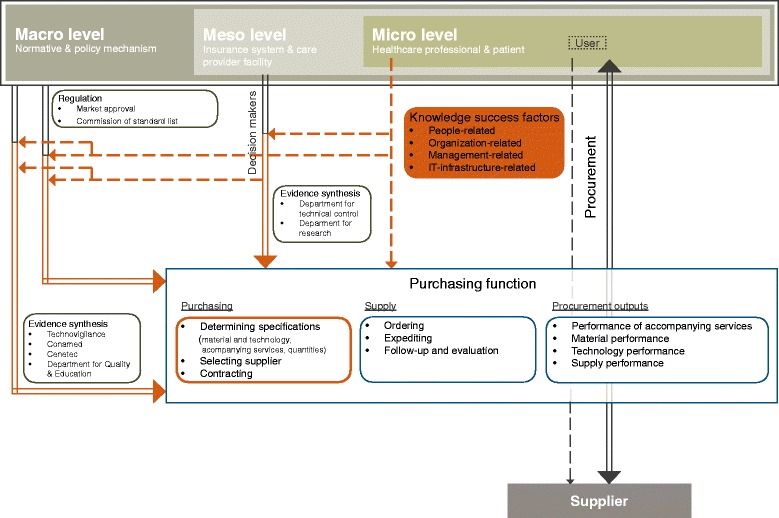
Research approach model

Our research approach is based on a working framework presented in Fig. 1, which is guided by two considerations (i) procurement supports healthcare delivery and (ii) procurement decision-making is knowledge sensitive.

First, we defined three healthcare delivery levels based on the healthcare delivery model [30]: 1) macro (normative and policy mechanism); 2) meso (insurance system & care provider facility); and, 3) micro (orthopaedic specialist and patient). Differentiating between these levels is a crucial aspect of our research because public procurement in Mexico and procurement decisions take place at the meso level and not at the micro level. The user is employed by the social security institute or ministry of health and has little autonomy during procurement decision-making in respect to select a medical device. This differs from other healthcare systems where users are self-employed and the procurement mechanism used by healthcare providers is independent of a central purchasing function [31].

Second, we explain procurement based on the supply link framework [32] and embed it along the three healthcare delivery levels. Procurement has three main actors: supplier; procurement administration (purchaser); and, internal customers (at the meso level) and users (at the micro level). The interaction between the main actors is shown by arrows and defined by (i) procurement (administrator or agent) and internal customer or user, and (ii) procurement and supplier.

Third, we implemented the four knowledge management dimensions [11] as the underlying concept of this research approach and used them as orientation to analyse factors of managing knowledge (healthcare delivery levels), to assess the role of knowledge from clinical procedures and in relation to procurement, and to identify findings having the ability to improve managing knowledge.

### Research method

The study was based on: (i) semi-structured interviews with healthcare system stakeholders that represented macro and meso levels (Group 1) to analyse how knowledge of clinical procedures is managed among the knowledge management dimensions; and (ii) semi-structured interviews with orthopaedic specialists who represent the micro level (Group 2) to assess the role of knowledge from clinical procedures and in relation to procurement of orthopaedic medical devices.

### Rationale and validity of selected research method

We chose this approach because a quantitative approach would not have given us enough data and because there were so few prospective participants representing the macro level and low-to-moderate number of prospective participants representing the meso level.

To ensure validity and reliability we used several strategies. First, during interviews we probed deeply to uncover attitudes and open up new dimensions of a problem, and to urge the stakeholder to describe their personal stake in the process. Secondly, we triangulated data by defining a heterogeneous sample of stakeholders per group, and finally, we used different interview guides (described in “data collection”) that we pre-tested with few stakeholders from Mexico.

### Study population and participant selection

We interviewed 48 people and their composition is presented in Table 1.

**Table 1:** Extraction of interview guide questions

Group 1	
Q1	On which organizational levels did Mexico achieve the required quality assurance of already established programs? Please explain how.
Q2	Which organizational level manifests the biggest barrier to translate efforts of quality assurance into results?
Q3	Please describe your opinion on quality assurance and clinical efficacy as contributing elements for the provision of medical devices?.
Q4	Please describe how clinical data from the clinical practice level is transferred back to the institutional level and how to the national level?
Q5	Please describe the general consciousness of Mexican stakeholders for the contribution of clinical evidence to their practice.
Q6	On which organizational levels or specific areas do you observe weaknesses with regards to the consciousness?
Q7	Other countries say that it is a challenge to assure clinical effectiveness of a medical device without the support of clinical evidence? Only product safety is not sufficient for high-risk medical devices. Based on which attempts or programs Mexico tries to manage this situation.
Q8	Please describe what this means for the clinical practice and outcome of the patient?
Q9	What actions are needed to improve this situation?
Group 2	
Q1	What should be the relation of the orthopaedic surgeon and the procurement of medical devices?
Q2	Please describe your role and knowledge in terms of the procurement of medical devices?
Q3	Please describe what this means for yourself as surgeon who takes over the responsibility for the clinical outcome of the patient?
Q4	In Mexico it is common practice to procure the majority of medical devices through tenders and to award based on the best price (respecting its listing in cuadro basico). What is your experience on that?
Q5	Please describe how you perceive the outcome of the procurement in terms of your clinical practice, the quality of supplier service, and the intrinsic quality of the product?
Q6	How is clinical evidence and clinical data considered?
Q7	Please describe how long you can stick to the same implant system in your public institution. Please distinct between trauma and reconstruction devices?
Q8	Please describe what this means for the clinical practice and outcome of the patient?
Q9	What actions are needed to improve this situation?
Q10	How do you currently obtain sustainable information on clinical safety of a medical device?

We identified and recruited participants for interviews by (i) searching listings from the ministry of health and industry for orthopaedic HRMDs, national academic experts, orthopaedic specialists, organisations, hospitals, and institutions to identify potential interviewees and (ii), we asked interviewees to recommend other stakeholders. We based the sample on two criteria: (1) recruit a heterogeneous sample across different stakeholders; and (2) stakeholder being involved in or familiar with regulations of medical devices, healthcare delivery of medical devices, procurement and provision of medical devices in Mexico. Sampling was rooted in a maximum variation strategy [33, 34].

### Data collection

The study was done in Mexico (Federal District and State of Mexico). In this area the concentration of arthroplasty surgery across the country and the representation of important government officials or key stakeholders of healthcare providers is high. In Mexico, healthcare providers belong to an institution of the social security sector (IMSS, ISSSTE, PEMEX, SEDENA, MARINA) that operate on national level or to the ministry of health (Seguro Popular de Salud, SEDS, Programa IMSS-O) [35].

We approached prospective interviewees between February and March 2015 them by email or phone. Before we invited them to an interview, the principal investigator talked or wrote to them. Interviews took place at the office of the interviewee or at a place the interviewee selected (e.g. conference room at work).

Interviews averaged 23 min (min = 18 min, max = 35 min). Interviewees had a choice of being interviewed in Spanish or English. We used a file naming system and anonymised interviewees by generating a list of archival numbers. The principal investigator interviewed Group 1 participants and some Group 2 participants. A research assistant interviewed the rest of the participants from Group 2. Of the 48 interviews we conducted, 96 % were face-to-face, and 4 % were phone interviews. We audio recorded all interviews and transcribed them with F5 software [36]. The principal investigator and two assistants transcribed the interviews, and the principal investigator reviewed them again. The interviewers used semi-structured interview guides including open-ended questions that encouraged interview participants to freely describe their opinions, thoughts and experiences (Table 2). Participants were not compensated monetary or otherwise.

**Table 2:** Composition of participants

Expertise of participant	Group 1	Group 2
	n	n
Total	25	23
Macro level		
Regulation	4	0
Evidence synthesis	6	0
Orthopaedic association	3	0
Other expert	2	0
Meso level		
Institution	4	0
Care provider facility	6	0
Micro level (orthopaedic specialist employed by different institutes)		
Social Security – IMSS	0	8
Social Security – ISSSTE	0	4
Social Security – PEMEX, SEDENA	0	3
Ministry of Health	0	8

*IMSS* Instituto Mexicano de Seguro Social (Mexican Institute of Social Security); *ISSSTE* Instituto de Seguridad y Servicios Sociales de los Trabajadores del Estado (Institute of Social Security and Services for State Workers); *PEMEX* Petróleos Mexicanos (Mexican Petroleums); *SEDENA* Secretaría de la Defensa Nacional (Secretariat of National Defense)

### Data analysis

We used our research approach model as a working framework and opted to analyse the findings by the four knowledge management dimensions because in this way, we were able to describe the connectedness and interaction between the actors directly or indirectly involved in procurement based on knowledge-related factors. Other research approaches concerned with knowledge management have been used as well but for different research questions or approaches [37]. We iteratively analysed the content of all interviews [33] in MAXQDA software version 11 [38] and to systematically inferred interdependencies between the experiences and opinions of stakeholders. First, we closely read each transcript (data orientation) during initial coding. Second, we clustered codes for similar themes and interrelated concepts (data reduction). Third, we revised our list of themes, improved codes and clustering if necessary, and clarified ambiguous statements (data display). Lastly, we drew on the themes we identified as deficiencies in the role and management of knowledge (conclusion drawing). The principal investigator analysed all data. Table 3 provides an extraction of relevant statements.

**Table 3:** Extraction of relevant quotations

Themes	Illustrative quotations	Interviewee
People-related factors		
Leadership	“At the strategic or functional level they define and develop and promote an idea and there is a disposition but after that they are failing with the implementation.”	Group 1, Macro – International Expert O.2._201503101730_MEX
	“There was once, in the past two administrations, during president Fox and president Calderon, very interesting quality assurance strategies for all public institutions. Again, mostly based on interpersonal quality and what the different institutions and the different facilities… what they achieved in terms of quality… depended a lot of the interest of particular clinical groups.”	Group 1, Macro - Evidence synthesis O.2._201502231200_MEX
	“So in Mexico we have the problem that they don’t talk to each other, they don’t understand each other and there is no governance… so that the way how they solve the problem is rather voluntary than an organizational or systemic matter.”	Group 1, Macro - Evidence synthesis O.2._201502271200_MEX
Knowledge competence	“The research culture here in Mexico is unfortunately very low in comparison with the culture of other north American or European countries…”	Group 1, Macro – Society O.2._201503191830_MEX
	“We still don't have a clear consciousness of the importance of implementing quality assurance measures at the system level. Quality concerns are concerned mostly of few groups within institutions. So I think the big challenge, the first initial challenge would be to develop a better consciousness of the importance of continuous improvement. This is I think the main challenge.“	Group 1, Macro - Evidence synthesis O.2._201502231200_MEX
	“… sometimes we receive drugs of very good quality. But sometimes we receive very bad quality because procurement doesn’t focus on this… As long as a drug passed the requirements of the health regulation of COFEPRIS there is a market…”	Group 1, Macro - Evidence synthesis O.2._201502271200_MEX
Knowledge sharing	“The doctors don’t always accept to provide this information. Let’s think about health records. This is really a problem that the doctors use them correctly… the information that is collected is little reliable.”	Group 1, Macro – International Expert O.2._201503101730_MEX
	“The problem is that many don’t fill in the type of incident … they don’t provide the name of the product. Therefore we cannot make a match and process the complaint adequately…”	Group 1, Macro - Evidence synthesis O.2._201503120900_MEX
	“… nobody notifies about adverse reactions in this country…”	Group 1, Macro - Evidence synthesis O.2._201503091215_MEX
Mutual learning & skills	“… they are still duplicating their efforts… but what is difficult to change is the burocratic territory of each institute and there is no incentive that could motivate them to focus on a common purpose… an therefore they make what they can but not always coordinated…”	Group 1, Macro – International Expert O.2._201503101730_MEX
	“I believe that we have to improve the quality … conceiving a better interrelation between COFEPRIS and other federal units of the secretariat of health.”	Group 1, Macro - Evidence synthesis O.2._201503091215_MEX
	“Because we don’t have, neither the resources and very probably we don’t have the expertise needed to follow up and to organize this kind of interventions.”	Group 1, Macro – Society O.2._201503232000_MEX
Organization-related factors		
National processes or structures	“… apart of adverse events there is no intermediate information available. So there is a lot of information that we loose… as surgeon you are very limited with regards to access information…”	Group 1, Macro - Evidence synthesis O.2._201503091215_MEX
	“I believe the weakest area is the federal with COFEPRIS and the strongest area is the Consejo de Salubridad General by means of CENETEC which is step by step better involved in the evaluation of medical technologies in a broad sense.”	Group 1, Meso - Institution O.2._201503121800_MEX
	“The problem is that no one makes a follow-up of the output of results. Recently the secretariat of health has started to establish an evaluation system of the performance of hospitals.”	Group 1, Macro – International Expert O.2._201502240930_MEX
Organizational processes or structures	“They provide us with some type of report, they inform us in general about number of prosthesis and patients… but we don’t receive more information.”	Group 1, Meso – Institution O.2._201502241600_MEX
	“Further many federal units have different organizational structures so that this makes the situation even worse… they are heterogeneous so that en some units they are well organized… but in others there doesn’t even exist such an organization to adapt specific programmes…”	Group 1, Meso – Hospital O.2._201503171700_MEX
	“When you ask what is the number of intra hospital infections that we have in Mexico nobody can provide you with a general number… this is something that haven’t been established in Mexico.”	Group 1, Macro – International Expert O.2._201502240930_MEX
Management-related factors		
Strategy	“… programmes are established, they are effused like documents to be used but rarely there is a control if these programmes are realized… especially at the level of the secretariat of health… There is a deficiency beginning at the central legal level up to the state level where there are no adequate strategies to implement a program to improve quality.”	Group 1, Meso – Hospital O.2._201503171700_MEX
	“The healthcare systems remains in the 21st century or migrated back to the 20th century. What I want to say is that this system is focusing to cover crises, episodes, but does not attend patients.”	Group 1, Macro - Evidence synthesis O.2._201502271200_MEX
	“…we are not using the information. We are collecting it and we are organizing it, we have the conditions to use it at very different levels, at the clinical level, at the top management level, but we are not using it. ”	Group 1, Macro - Evidence synthesis O.2._201502231200_MEX
Goal	“The problem is that we are affiliating people and little by little starting to guarantee regular access to comprehensive service. Unfortunately the overall quality of the services that are being provided is still very low especially at the ambulatory level. So, it is good, that we are expanding coverage, but we need to expand coverage with quality. If not, we are misspending the resources we have mobilized. ”	Group 1, Macro - Evidence synthesis O.2._201502231200_MEX
	“Recently a new epoc has started where we can say what are the palliative aspects that impacted … The famous collateral damages or additional expenses, or I had to keep the patient hospitalised longer because I could not operate him because the implant failed.”	Group 1, Meso – Hospital O.2._201503130830_MEX
	“If you go to a hospital and you want to certify it and you ask them “Is there technical support for cardiotocography in the urgency unit”, the answer may be yes but no one would ever ask if they also know how to interpret the data.”	Group 1, Macro - Evidence synthesis O.2._201502261200_MEX
Measurement	“… the interesting thing is that there were no indicators for the number of prescriptions that are aligned with the clinical guidelines.”	Group 1, Macro - Evidence synthesis O.2._201503091215_MEX
	“And so, here most time the evaluations stop evaluating the existence of a product…”	Group 1, Macro - Evidence synthesis O.2._201502261200_MEX
	“…The infrastructure is limited and this is a serious problem because there is interest … but also the money is an important limitation…”	Group 1, Macro - Evidence synthesis O.2._201502261115_MEX
Information technology-related factors		
Infrastructure	“… There are two problems that I can identify: One is the absence of basic information systems … our information is in general not systemised.”	Group 1, Macro – International Expert O.2._201503101730_MEX
	“Our registries are not complete, they are not reliable because not everything is registered. Therefore it is an idea of numbers… but a precise number requires a good registry with a very good systematisation…”	Group 1, Meso - Hospital O.2._201502251245_MEX
	“…There might exist a lot of data in the different social security systems or within the same system but they are not in a single database”	Group 1, Macro - Evidence synthesis O.2._201502250830_MEX
Applications	“And one of the more serious problems is the information system. They are not based in patients, they are based in medical consultations, in hospitalization…”	Group 1, Macro - Evidence synthesis O.2._201502271200_MEX
	“In some institutes … they do have an electronic health record, but it is another deficiency that our country was not able so far to consolidate the electronic health records on national level…”	Group 1, Macro - Evidence synthesis O.2._201502261200_MEX
	“… it is very difficult in the big hospitals and not all do have a health record, there are big hospitals that have health records but they don’t use it.”	Group 1, Macro - Society O.2._201503191830_MEX
Factors related to the role of knowledge from clinical procedures		
Relation of orthopaedic specialist and procurement	“… because the procurement process here in … is rather confidential, not all doctors participate, and sometimes it is very superficial so that it is only about affirmative or negative, but … often someone like a doctor doesn’t participate.”	Group 2, Micro - Social Security O.2._201502241300_MEX
	“They don’t take into consideration the surgeon to take decisions because often the administrators decides and they buy things that no one uses.”	Group 2, Micro – Ministry of Health O.2._201503121730_MEX
	“… this decides the head of the department together obviously with the hospital director and … it is like a rather private situation…”	Group 2, Micro - Social Security O.2._201503121830_MEX
Knowledge informed decision-making	“…no because it is expected that they (COFEPRIS) have taken care of it, they have test it and everything is good and this is not true, many time not.”	Group 2, Micro - Social Security O.2._201503241330_MEX
	“… many times the decision-making is based on the material type and economical aspects or the cost of these implants.”	Group 2, Micro - Social Security O.2._201503181300_MEX
	“No, I think it is very bad (information flow between micro and meso level) what is exchanged between us because we have requested a meeting between the people of the Seguro Popular and us to explain which material is good and adequate for the patients. But this has never taken place…”	Group 2, Micro – Ministry of Health O.2._201503131230_MEX

## Results

We found that knowledge is not necessarily generated and managed efficiently enough to support procurement of orthopaedic HRMDs. Generally, interviewees thought this is a problem at the meso level and related to the dimensions “people” and “organisation”. Table 4 shows the relevance of inadequately managed knowledge for all four dimensions, at the macro, meso, and micro level.

**Table 4:** Relevance to strengthen management of knowledge for all four dimensions and healthcare delivery levels

Dimension	Macro level	Meso level	Micro level	Total
People	++	+++	++	++(+)
Organisation	++	+++	++	++(+)
Management	+	++	+	+
Information technology	+	++	++	+(+)
Total	+(+)	++(+)	++	++

+++ very relevant ++ moderate relevant + relevant

The problems that were associated at the macro, meso and micro levels, for the various knowledge-related factors, influence the role of knowledge from clinical procedures and in relation to procurement of orthopaedic HRMD. The results of our study show that this leads to procurement decision-making that is insufficiently informed by this knowledge and thus negatively influences the provision and use of orthopaedic HRMDs. In Table 5 we summarize themes that describe these problems based on the knowledge management dimensions and the role of knowledge for procurement.

**Table 5:** Summary of management and role of knowledge

Topic	Identified themes
Dimension “people”	• Absence of a mutual learning culture specifically for HRMDs. • Inadequate knowledge sharing culture to manage complaints. • No sustainable commitment to clinical knowledge-informed quality assurance programmes. • Need to engage people in generating knowledge. • Organisations unable to generate knowledge. • Uncertainty how to apply knowledge correctly • Failure to identify the relevance of post-market surveillance data.
Dimension “organization”	• Absence of structures to improve handling and management of complaints and adverse events across departments. • Absence of structures to obtain adequate information of data from clinical procedures.
Dimension “management”	• Opportunities to develop strategies that merge the interests of the different sectors to achieve federal knowledge goals. • Need for improved exchange of information between federal units and insufficient to create synergies. • Opportunities to implement strategies that can adequately measure e.g. the clinical performance of MDs.
Dimension “information technology”	• Insufficient implementation of electronic patient data collection and records. • Lack of infrastructure for collecting national post-market surveillance data. • Need for an application that monitors performance of MDs in clinical use.
Role of knowledge	• Rigid evaluation criteria like demand calculation. • Lack of orthopaedic experts on decision-making committees. • Importance of lowest acquisition price. • Feedback loop on performance of HRMD between users and administrators.

We divided our findings into three levels: (i) dimensions of managing knowledge, (ii) the role of knowledge from clinical procedures and in relation to procurement of HRMD, and (iii) opportunities to improve the situation.

### Dimensions of managing knowledge

#### People-related factors as barriers

One of the management theory expectations that can be applied to this analysis is that if the culture of knowledge is well established among the actors in a healthcare system, knowledge can be adequately managed. However, the results of this study show that knowledge is not adequately managed at the meso level. To a lesser degree, this is also true at the macro and micro levels. Many stakeholders reported that there is not enough knowledge leadership (e.g., guidance and direction in using knowledge) or competence to ensure knowledge will be efficiently managed and used. The culture of knowledge sharing and mutual learning is underdeveloped. We found a number of themes in the transcripts that described the effect of people-related factors on knowledge management, related to (i) leadership, (ii) knowledge competence, (iii) knowledge sharing, and (iv) mutual learning.

Leaders direct the people who generate or manage knowledge. Some stakeholders emphasized the strong influence that key leaders have on directing and implementing knowledge sharing initiatives, or on continuing strategic quality assurance initiatives. Initiatives are often discontinued or disrupted when the initiator moves on to other tasks or passes the responsibility to others.

*“[T]here was once, in the past two administrations… very interesting quality assurance strategies for all public institutions… what they achieved in terms of quality… depended a lot of the interest of particular clinical groups.”*

(Group 1, O.2._201502231200_MEX)

Knowledge competence and sharing allow people to integrate knowledge effectively into their work. Some participants mentioned this because in the area of clinical research and investigation of orthopaedic speciality little is published by Mexican orthopaedic specialists in scientific journals and they related this to lack of interest, a general weakness of the medical education system, or the work framework for medical specialists in public hospitals. For orthopaedic specialists in other countries, the publication track record is important for their career paths.

*“[T]here is little culture to publish scientific work… few are really dedicated to this… because of a missing focus during the medicine study and because of high workload at the public institutes… and so it is difficult to focus on research.”*

(Group 1, O.2._201503191330_MEX)

Some participants reported that care providers from the secretariat of health partially manage knowledge more efficiently than did social security systems. For instance, The National Institutes of Speciality (e.g., the National Institute for Rehabilitation) have a more developed system for managing and using knowledge than the regional hospitals of the secretariat of health. We found that this is a consequence of various people-related factors.

*“…[t]his is all a process and we are all at different levels and a lot of what can be achieved in each process can be related to the interest of research groups intra- or extra-institutional… but we are not all at the same level… some haven’t started yet.”*

(Group 1, O.2._201502251245_MEX)

#### Organisation-related factors as barriers

Efficient management and use of knowledge is facilitated if actors effectively use processes and structures in a healthcare system. But the results of this study show that knowledge is often inadequately managed at the meso level. This is less of an issue at macro and micro level. The formal processes and structures are insufficient to facilitate efficient management and use of knowledge because post-market surveillance data is inadequate. Thus, information flows insufficiently, knowledge spreads poorly, and there is little synergy created by processes that run in parallel. Interview participants described organisation-related factors that, in their view, contributed to the failure of national organisations to manage knowledge adequately, especially on a process or structural level, such as the organisational processes or structures of social security systems or care providers of the secretariat of health.

Many participants pointed out that current formal processes and structures make it difficult to collect adequate post-market surveillance data because they inadequately integrate knowledge about clinical procedures.

*“… [t]he principal weakness of the Mexican system is at the post-commercialization…”*

(Group 1, O.2._201502261115_MEX)

Clinical data collection starts with clinical procedures and needs to be established, e.g., a post-market surveillance system. Some interviewees said that current processes and structures do not connect the meso and micro levels well enough; data collection is inconsistent, so clinical procedures do not generate adequate knowledge.

*“…[a]part of adverse events there is no intermediate information available. So there is a lot of information that we lose… as a surgeon you are very limited with regards to access information…”*

(Group 1, O.2._201503091215_MEX)

*“[B]ut you don’t follow up (clinical cases). You know when you follow up, this is when there is any complication…”*

(Group 2, O.2._201503111600_MEX)

Some participants claimed that medical specialists often have restricted access to information that coordinates meso level actors from departments like administration or research and quality. Medical specialists rely on a limited set of data to perform clinical procedures or research, and there are no monitoring processes for following up clinical cases over the long-term.

*“[T]hey provide us with some type of report, they inform us in general about the number of prosthesis and patients… but we don’t receive more information.”*

(Group 1, O.2._201502241600_MEX)

Further, participants noted that formal processes and structures that intended to improve quality did not allow actors in a health system to create synergy with other actors running in parallel. They also noted that these are poorly coordinated because the health system is fragmented and segmented. Creating synergies improve outcomes of single processes or strategies like national programmes and initiatives.

*“…[t]hey are still duplicating their efforts… but what is difficult to change is the bureaucratic territory of each institute and there is no incentive that could motivate them to focus on a common purpose… an therefore they make what they can but not always coordinated…”*

(Group 1, O.2._201503101730_MEX)

For instance, formal processes and structures for the management of complaints related to the use of HRMD at the macro level: The National Commission for Medical Arbitration (CONAMED) receives complaints from patients about service attention of care providers, and the Department of Technovigilance of the Federal Department of Health and Human Services of Mexico (COFEPRIS) also documents HRMD complaints but on the level of e.g. adverse events (e.g. metal debris cause damage to bone reaction; bone cement insufficiently attaches to cemented implant surface; pelvis cap anchoring technology leads to early loosening of implant) [39] and reported by the physician or medical device supplier. However, CONAMED and COFEPRIS have no processes in place to share and mutually learn from these complaints.

#### Management-related factors as barriers

Knowledge strategy, goals, and measurement (e.g., knowledge control, measurement criteria, performance indicators) provide direction to actors in a healthcare system. Actors can then manage and use knowledge efficiently and follow-up on strategies, thereby increasing the effectiveness of their strategies and goals. Some stakeholders felt they were not given adequate direction. This was moderately prevalent at the meso level, but rare at the macro and micro levels. Our analysis revealed several themes where stakeholders related the failure to manage knowledge adequately to management-related factors, since these failures were observed in strategies, goals, and measurement of (i) federal units, (ii) care providers of the secretariat of health, (iii) social security systems, and (iv) healthcare professionals.

The participants reported that it is difficult to fulfil the national goals in the health system since the coordinating role of the ministry of health is weak, particularly in the relations with the social security organizations and the health systems in the sovereign states in the country. Thus, care providers may not apply national strategies because they are not obliged to.

*“…[p]rogrammes are established, they are effused like documents to be used but rarely there is a control if these programmes are realized… There is a deficiency beginning at the central legal level up to the state level where there are no adequate strategies to implement a program to improve quality.”*

(Group 1, O.2._201503171700_MEX)

Some participants explained that strategies are sometimes based on goals but are still disconnected from clinical procedures or rely on other data that may not fully represent clinical needs. For example, in recent years, many clinical guidelines have been written and introduced. Stakeholders who know clinical procedures complain that the goal of introducing so many clinical guidelines took precedence over developing strategies to benefit clinical procedures and processes.

*“[A]nd if we had focused to develop clinical guidelines for a limited number of diseases and have made the implementation strategy more carefully with measurements and incentives we would have another scenario… Now the problem is big because I don’t know how the clinical guidelines will be updated…”*

(Group 1, O.2._201503101730_MEX)

Federal units do not have well-established strategies to effectively collaborate with each other, as seen with CONAMED and COFEPRIS.

*“… [C]OFEPRIS… Consejo de Salubridad General… Cuadro Basico… CENETEC… and these four federal entities have been quite disconnected…”*

(Group 1, O.2._201503091215_MEX)

There was a similar problem at the meso level. Departments for research and quality look at HRMD failures through the lens of material specification or technology. They focus strongly on the indications of the standard list for HRMDs “Cuadro Basico”, but do not seek to gain knowledge from the observations that orthopaedic specialists generate during clinical procedures. These observations might include other types of product failures like anatomical aspects of HRMDs, steps in inserting or removing a HRMD, or special components of the instrument that cost clinicians a lot of time.

#### Information technology-related factors as barriers

Infrastructure and applications create the technical environment where knowledge is managed within and between the different levels of healthcare delivery. Information technology is an important aspect to transfer and process knowledge [3]. Some interviewees pointed out inadequate knowledge management being moderately prevalent at the meso and micro levels, and less prevalent at the macro level. Technological support is less efficient at the meso and micro levels, where administrators and healthcare professionals operate. The problem consists of information being insufficiently collected and analysed. For several themes, interviewees associated the failure to manage knowledge adequately with the absence of technological solutions.

Some stakeholders pointed out that current applications are not set up to run analyses of interest, like determining the performance of HRMDs in use. They said that there is limited infrastructure for sharing clinical data with healthcare professionals or other care providers within the same public sector. Some explained that insufficient infrastructure and failure to systematise data makes it hard to merge data from different public sectors.

*“…[T]here are two problems that I can identify: One is the absence of basic information systems … our information is in general not systemised.“*

(Group 1, O.2._201503101730_MEX)

For instance, CONAMED uses a web-based system to collect information about clinical incidents, which are reported mainly by patients. Aligning this system with databases from the social security systems would increase the knowledge that could be drawn from these data. A few stakeholders reported that one of the social security sectors is working with CONAMED to do this. There are few adequately developed applications that collect and store patient data. Applications that monitor the performance of HRMD are incomplete or unavailable.

*“[I]n some institutes … they do have an electronic health record, but it is another deficiency that our country was not able so far to consolidate the electronic health records on national level…”*

(Group 1, O.2._201502261200_MEX)

### Role of knowledge from clinical procedures and in relation to procurement of orthopaedic HRMD

Orthopaedic HRMDs are procured in Mexico through an administrative process that relies on standardized regulations to consolidate purchase power. These are mainly based on tender processes that regroup different purchases to increase purchasing power and negotiate better prices from suppliers. The results of this study show that knowledge from clinical procedures is insufficiently integrated into procurement decision-making. Many stakeholders thought this was caused by standardized procurement regulations and problems with knowledge exchange between orthopaedic specialists and administrators or between management levels of care providers. We found a number of themes, which described the very small role played by knowledge of clinical procedures.

A number of interviewees indicated that orthopaedic specialists are insufficiently involved and that procurement applies rigid evaluation criteria like demand calculation based on consumption history, and conformity controls based on technical or material specifications. However, stakeholders are very interested in procurement decision-making that integrates the orthopaedic specialist.

*“[T]hey don’t take into consideration the surgeon to take decisions because often the administrators decides and they buy things that no one uses.”*

(Group 2, O.2._201503121730_MEX)

Some interviewees claimed that when procurement did involve medical specialists, they were often not in orthopaedics or were unfamiliar with local clinical needs. Hiring of responsible staff that could contribute to improving the outcome of procurement decision-making was inconsistent.

Other respondents stated that decision-making was strongly influenced by the lowest acquisition price. In our first study informants already noted this. Orthopaedic specialists attribute their inferior role in decision-making to the acquisition price factor.

*“…[i]t is a straight situation of money, this is the only thing that really matters…”*

Group 2, CP_O.2._201503311600_MEX

Another theme that some participants emphasized was the formal complaint management processes. They noted that these did not influence procurement decision-making enough because complaints were not well-managed. For example, a group of orthopaedic specialists repeatedly received sub-standard quality of orthopaedic HRMDs, even after they had submitted formal complaints. They were eventually able to change their local procurement practices to incorporate knowledge from clinical procedures and post-market surveillance data of HRMDs. This change was only possible because the specialists insisted on escalating their complaints to upper-level management in their social security system, over several years. This situation seems exceptional. According to stakeholders of other healthcare providers, the problem of receiving sub-standard quality of HRMDs and services has not been solved.

*“[L]et’s say that I think that these companies can’t afford to manage the volume of the hospital and for example the other day I wanted to implant a femoral cup size 52 but I only had available size 50 and 54 and so I had to implant the cup size 50.”*

Group 2, O.2._201503181600_MEX

### Opportunities to improve the situation

Based on the first two objectives of this study we depicted which knowledge-related factors may lead to a situation of inconsistently generated knowledge of orthopaedic clinical procedures and in the context of procurement. The third objective of our study aimed to identify opportunities that may improve this situation by drawing on the findings of the previous two objectives and by asking interviewees what they believe is needed to improve the situation.

Many factors that we identified during the thematic analysis are related directly or indirectly to technological aspects, which we found are insufficiently developed.

*“[W]ell, I believe it is a matter of stewardship… of the ministry of health where clinical evidence should be regulated, from the clinical guidelines, the eligibility of goods and their regulation, monitor the clinical practice and provide feedback; overall, feedback… regulated for the private and public sector”*

Group 1, O.2._201503091215_MEX

*“[I]n some institutes, in some hospital centres of medical third level attention… there, electronical patient dossiers exist… however, and this is another deficiency, our country was unable to consolidate them at a national level, as it was proposed by the previous administration.”*

Group 1, O.2._201502261200_MEX

For public procurement in Mexico we believe that there is an opportunity to develop an action plan how to improve the management of systems’ knowledge across all social security institutes and ministry of health. Options of information technology may provide a basis in order to improve the intersections that procurement has with the knowledge environment (areas and activities relating to evidence and knowledge synthesis).

Procurement is an administrative area that is influenced by four principal aspects: Policy mechanisms and regulations; key procurement actors; degree of procurement centralization; and, criteria used to make procurement decisions [27]. In Mexico, public procurement practices are highly standardized and key procurement actors belong to the meso level and rarely to the micro level. The results of this study show that opportunities to improve the current situation were often associated with “key procurement actors” or “criteria used to make procurement decisions”.

*“[T]o improve we have to destroy the chains that limit the genuine commitment of doctors to look for a system, an implant of a quality; his decision nowadays is rather next to financial or administrative decisions. I believe we have to give greater emphasis to the doctor who is finally the user of implants…”*

Group 2, O.2._201502251340_MEX

*“[F]or me, at least in my institute that the technical advise is taken again into consideration…”*

Group 2, O.2._201502271600_MEX

*“[T]here should be communication of the directive of the sector towards the doctors… it should therefore integrate heads of departments and between them reach a consensus and a way to define the required materials to treat patients.*

Group 2, O.2._201503131230_MEX

The mechanism of public procurement in Mexico may not allow to actively integrating users in decision-making but there are opportunities to better integrate user knowledge. For instance, monitoring relevant aspects of clinical procedures that are important to assure the healthcare worker and patient safety by modifying the needs assessment strategy in the course of upcoming tenders.

## Discussion

In the Mexican Healthcare System and on behalf of the Ministry of Health many changes have taken place especially since 2006, such as comprehensive reforms to improve the health system [40–42], sectorial health programmes or research to improve quality across various dimensions [29, 43–46]. This is an important strength of the system because it is frequently concerned with situations lacking the ability to make progress in their performance.

Based on our findings it was evident that stakeholders in Mexico recognize that knowledge is an important resource but they are not able to manage it effectively and efficiently. The examples provided by the interview participants lead to important factors that trigger this situation and which we identify as information technology-related factors. The knowledge-related problems reported by interviewees focused strongly on “People” and “Organisation” but are connected to information-technology. For instance, participants referred to problems of systematic databases, not using synergies and being unable connecting the variety of systems’ knowledge. Without adequate infrastructure and applications to manage big data across the different healthcare delivery levels knowledge-related problems summarized in Table 4 and 5 cannot be solved adequately. In Mexico, policy makers have already identified the added value of information technology supporting procurement. For instance, the introduction of “compranet” [47] as application that provides transparency in respect to expenditures and awards of public tenders, and which operates mainly on the meso and macro level. We did not identify applications established that are based on a systematic approach to manage knowledge from clinical practice and connecting to procurement.

Overall we found that in Mexico the knowledge environment influences procurement regulations and practices of orthopaedic HRMDs in the following ways: 1) deficiencies in the healthcare system’s ability to manage knowledge of clinical procedures efficiently; and 2) deficiencies in the management of knowledge from clinical procedures and post-market surveillance data as it directly relates to procurement. Analysing knowledge-related factors, guided by considering the four knowledge management dimensions, lead us understand which factors trigger ineffective and inefficient knowledge management. The findings of this study point out knowledge-related opportunities for procurement practices of orthopaedic HRMDs in Mexico.

We found that the ability of procurement administrators or agents may improve when knowledge of orthopaedic clinical practices is adequately integrated in decision-making processes [27]. Procurement administrators or agents are concerned with providing the right quality of the purchased products (manage product complexity) and accompanying services (prevent commercial uncertainty) [48, 49]. Studies focus on knowledge gaps about buyer-supplier relationships [50] but not with knowledge-related factors influencing procurement and purchasing of HRMDs.

In our study we found that factors triggering the ineffective and inefficient use of knowledge can be associated with poorly developed technological solutions at the level of clinical procedures. We believe that there is an opportunity in managing knowledge in the field of orthopaedic HRMDs by adequately applied information technology solutions. Studies are concerned with health information management and technology and how it can be utilized to improve important outcomes and overall quality of care in different health care settings [51–54]. The interest in knowledge-related topics in healthcare systems is often focused on clinical informatics to promote patient care and safety like, for example, clinical decision-making. In this context, many studies report about eHealth solutions (managing single and aggregated health information for healthcare professionals, patients, and healthcare consumers), and applying it in clinical decision-making [5, 7, 55, 56] like, for example, the use of electronic patient dossiers operating at both the clinician and patient level [57].

Further, managing big data becomes more relevant [58] and we found that the use of options supporting knowledge management in the field of orthopaedic HRMDs by information technology applications are promising [54]. In the field of orthopaedics many policy makers use already approaches of information technology to guide decision-making. Examples for this are national arthroplasty registries [59], and approaches that build on such arthroplasty registries like, for instance the “Orthopaedic Data Evaluation Panel” (ODEP) in the UK. ODEP is defined as a supporting decision-making instrument for procurement. ODEP rates implant survival data based on clinical information and clinical evidence. It represents a guideline for procuring orthopaedic HRMDs and is established by the National Institute for Health and Care Excellence of the UK. ODEP rates implant survival data based on clinical information and clinical evidence level, received from the “National Joint Registry” (NJR) of the UK. The NJR collects information on orthopaedic joint replacement surgery from clinical procedures, and monitors the performance of orthopaedic implants. Other healthcare systems like, for example, Germany and Switzerland, integrate information from arthroplasty registries into their quality agenda [60, 61]. Without using information technology applications to manage big data it would not be possible to inform procurement decision-making adequately with information and knowledge of clinical practice.

We found that knowledge-related factors influencing procurement practices are not a unique finding for Mexico and orthopaedic HRMDs. The results of this study are primarily relevant for Mexico but may also give impulsion to other health systems with an increase of centralized procurement, like for example: Collaborative procurement hubs (e.g. United Kingdom), and national or regional purchasing groups (e.g. France, Germany) [27, 50, 54].

## Limitations and avenues for further research

Our study has several limitations. First, even we have opted a sampling based on a maximum variation technique, we did not include (i) a larger number of stakeholders representing the meso level of different social security systems, and (ii) patients or representatives from rehabilitation centres to provide a broader range of attitudes of the micro level. Secondly, our ability to generalize the findings was limited as we only considered orthopaedic HRMDs. Third, attitudes of stakeholders from other states may differ from those of the State of Mexico and the Federal District. Fourth, we did not take a formal knowledge management approach to clearly differentiate, e.g., between knowledge management systems and information systems.

More research is needed to clarify some issues raised in this study. What programmes could be established to improve the contribution of clinicians to knowledge management practices? What do our findings mean for the national health budget? Answering these questions is imperative to improving the generation and management of knowledge about clinical procedures as it is related to the procurement of orthopaedic HRMDs in Mexico.

## Conclusions

We believe this is a novel investigation of knowledge-related factors that influence procurement and clinical procedures for orthopaedic medical devices in Mexico. We identified specific aspects of knowledge and related them to procurement practices, using orthopaedic HRMDs as our example, and showed how they are related with clinical practice.

We explored the perceptions of a range of healthcare actors around the topic of generating and managing knowledge for improved procurement processes of orthopaedic devices. We showed that knowledge is an important resource, identified factors along the dimensions of knowledge management and healthcare delivery levels that create barriers, and discussed them in the context of administrative processes. The deficiencies we identified should motivate researchers to further clarify the relationship between clinical procedures and administrative processes in the knowledge environment.

Stakeholders in Mexico recognize that knowledge is an important resource, but they are not able to manage it effectively and efficiently. A favourable approach would be when procurement administrators exchange more knowledge with orthopaedic specialists who have performed surgical techniques, know the clinical properties of implants, and are familiar with the services provided by suppliers (e.g., the condition of instrument sets and availability of implant type or size), to improve procurement outcome. Without adequate solutions of managing knowledge for orthopaedic services, procurement processes for orthopaedic HRMDs will remain sub-optimal. Mexico needs versatile solutions for the meso level and the federal level of the Mexican healthcare system so as to better analyse information and data from clinical procedures. Many of our findings can be attributed to poorly developed information technology aspects. Improving options of managing knowledge by information technology may positively influence the impact of procurement decision-making on clinical practice and improve the healthcare worker and patient safety in the long-term.

## Abbreviations

CENETEC, Centro Nacional de Excelencia Tecnológica en Salud (National Centre for Health Technology Excellence); COFEPRIS, Comisión Federal para la Protección contra Riesgos Sanitarios (Federal department of health and human services of Mexico); CONACYT, Consejo Nacional de Ciencia y Tecnología (National Council of Science and Technology); CONAMED, Comisión Nacional de Arbitraje Medico (National commission for medical arbitration); e.g., exempli gratia; etc., et cetera; HRMD, high-risk medical device; IMSS, Instituto Mexicano de Seguro Social (Mexican Institute of Social Security); IMSS-O, Programme of Ministry of Health for non-insured population living in specific states or areas: Instituto Mexicano de Seguro Social – Oportunidades (Mexican Institute of Social Security - Opportunities); ISSSTE, Instituto de Seguridad y Servicios Sociales de los Trabajadores del Estado (Institute of Social Security and Services for State Workers); MARINA, marine; NJR, National Joint Registry; ODEP, orthopaedic data evaluation panel; PEMEX, Petróleos Mexicanos (Mexican Petroleums); SEDENA, Secretaría de la Defensa Nacional (Secretariat of National Defense); SEDS, Servicios Estatales de Salud (State Health Services); WHO, World Health Organization.
